# Use and perceptions of Cannabidiol among individuals in treatment for opioid use disorder

**DOI:** 10.1186/s12954-024-01051-5

**Published:** 2024-07-17

**Authors:** Christopher Kudrich, Rebecca Chen, Yuan Meng, Keren Bachi, Yasmin L. Hurd

**Affiliations:** 1 Department of Psychiatry, Icahn School of Medicine at Mount Sinai, Addiction Institute of Mount Sinai, 1399 Park Ave, Room 3-330, New York, NY 10029 USA; 2 Icahn School of Medicine at Mount Sinai, New York, NY USA; 3 Department of Environmental Medicine and Public Health, Icahn School of Medicine at Mount Sinai, New York, NY USA; 4 Department of Neuroscience, Icahn School of Medicine at Mount Sinai, Addiction Institute of Mount Sinai, New York, NY USA

**Keywords:** Cannabidiol, CBD, Cannabis, Opioid use disorder, Methadone, Buprenorphine

## Abstract

**Background:**

Cannabidiol (CBD) is a widely available cannabis product with many claims as to potential health benefits including alleviating symptoms related to opioid use disorder (OUD). However, little is known as to how individuals with OUD perceive CBD, to what extent they may already be using CBD, and for what purposes.

**Methods:**

A survey was conducted among individuals receiving treatment for OUD at the Addiction Institute of Mount Sinai in New York City from July 2021 to August 2023. The survey consisted of demographic questions, questions about opioid use, CBD use, and perceptions regarding CBD. Statistical analysis using ordinal logistic regression was employed to compare perceptions between CBD users and non-users while adjusting for age and race.

**Results:**

Among 587 respondents, 550 completed the survey. Among all survey completers, 129 (23%) reported a history of using CBD for a variety of reasons including: anxiety (81, 62.8%), pain (65, 50.4%), sleep (63, 48.8%), depression (62, 48.1%), recreational purposes (32, 24.8%), or for other reasons (8, 6.2%). Of note, 22 (17.1%) respondents reported using CBD to control their addiction and 54 (41.9%) reported using CBD to ease opioid withdrawal symptoms. CBD users demonstrated more positive perceptions regarding its legality (β = 0.673, OR = 1.960, 95% CI [1.211, 3.176], *p* = .006), social acceptance (β = 0.718, OR = 2.051, 95% CI [1.257, 3.341], *p* = .004), and therapeutic potential compared to non-users. CBD users also had a more positive view of its potential future role in managing addiction (β = 0.613, OR = 1.846, 95% CI [1.181, 2.887], *p* = .007).

**Conclusions:**

This study highlights a significant association between CBD usage and progressive views regarding CBD among individuals with OUD, suggesting a growing interest in CBD as a potential adjunctive therapy for individuals in substance use treatment. Some patients are already using CBD for anxiety, pain, sleep, depression, or as a harm reduction intervention to control their addiction or for opioid withdrawal symptoms. These findings underscore the importance of integrating patient perspectives into future research and treatment strategies involving CBD in the context of OUD.

**Supplementary Information:**

The online version contains supplementary material available at 10.1186/s12954-024-01051-5.

## Background

Cannabidiol (CBD) has become a widely available over-the-counter (OTC) cannabis product. This has led to an abundance of CBD-containing dietary supplements with various unsubstantiated health claims [1] and an explosion of public interest in the use of CBD [2]. CBD is a non-intoxicating phytocannabinoid with a good safety profile [3] which is currently being studied for a range of medical uses including pain, anxiety, insomnia, and substance use disorders. Currently, there is only one FDA-approved CBD prescription medication, which is indicated for seizure disorders [4, 5]. However, an array of CBD products currently exist including oils, tinctures, plant material that is smoked or vaporized, capsules, and gummies [6].

The opioid crisis has brought attention to CBD as a potential non-addictive treatment for opioid use disorder (OUD). Such therapeutic potential was suggested in a double-blind randomized placebo-controlled study, where CBD at high doses (400 mg and 800 mg) reduced cue-induced craving and anxiety in abstinent heroin users [7]. Moreover, CBD was also reported to reduce chronic pain and reduce the need for opioid pain medication in chronic pain patients [8].

The perception and use of CBD in individuals with OUD is still not understood. In a prior survey study, among individuals with substance use disorders (SUDs), CBD use was not common. However, this research focused primarily on individuals with alcohol use disorder and only 12% (n = 58) of respondents were opioid users. Interestingly, those who were taking CBD reported improvements in pain, agitation, anxiety, and sleep [9]. These symptoms are common complaints among individuals suffering from acute or protracted opioid withdrawal, suggesting a potential therapeutic benefit of CBD [10]. Additionally, a review article on the potential of CBD as a harm reduction intervention concluded that CBD may be able to reduce drug craving and improve well-being, which could lead to better adherence and engagement in substance use treatment such as opioid agonist therapy [11]. Since CBD is widely available OTC, it is unknown whether individuals with OUD presently use CBD as a harm reduction intervention to better control their opioid use or withdrawal symptoms.

Emerging evidence suggests that CBD may hold promise as a future treatment for substance use disorders [12]. However, there is still limited data on whether patients undergoing treatment for OUD are using CBD, their reasons for using it, their perceptions of CBD, and their attitudes toward CBD as a potential future treatment for OUD. This study aimed to fill this gap by exploring the real-world use and perceptions of CBD among patients in treatment for OUD.

## Methods

### Design

This was a cross-sectional convenience survey study targeted at patients actively in treatment for OUD. Patients were in all stages of the disease including those in methadone maintenance, suboxone maintenance, detoxification, and rehabilitation. Survey participants were voluntarily recruited from clinical sites at the Addiction Institute of Mount Sinai in New York City. No incentives were provided to participants to complete the surveys. Surveys were administered from 07/15/2021 to 8/11/2023.

### Institutional review board approval and informed consent process

The study was reviewed by the Institutional Review Board of Icahn School of Medicine at Mount Sinai and was given exempt status. Participants were informed of the purpose, scope, length, exclusion criteria, and potential risks of the study via a consent form presented at the start of the online survey. The consent clarified that no identifiable information would be collected, and that the participant could stop the survey at any time. The name and contact information of the primary investigator and the contact information for the Program for the Protection of Human Subjects Office were also listed. All electronic data records were stored in a password-protected file on a secure server.

### Survey development

The survey was developed by clinicians and researchers at the Icahn School of Medicine at Mount Sinai using REDCap data collection software [13, 14]. Questions were based on prior surveys used in CBD research [15–17] and consisted of three sections. The first section included demographic questions documenting the age, gender, education, ethnicity, race, and employment status of the participants. The second section was a brief multiple-choice segment on opioid use history. The final section asked participants about their familiarity with, use of, and perceptions of CBD. Branching logic was employed, where responses determined which subsequent multiple-choice or Likert scale questions were displayed to the participant. The survey was primarily in multiple-choice format, with four questions providing an “other” option for additional responses. All questions underwent multiple rounds of review and editing by the clinicians and researchers before being finalized for the study respondents (Online Appendix 1).

### Recruitment process and survey administration

Flyers with a QR code that linked to the survey were posted at the clinical sites allowing participants to engage in the study independently. In addition to QR codes, research team members recruited the majority of participants through direct engagement using a convenience sampling method, with study personnel conducting face-to-face recruitment and providing participants with electronic tablet devices to complete the survey.

### Data collection and analysis

Data was collected using REDCap. Only completed surveys were used in the final analysis. Data was analyzed using REDCap reports and SPSS statistical software. Descriptive statistics were performed to analyze sociodemographic information (difference in age was analyzed using a t-test, while all other sociodemographic data and opioid use history was analyzed with Chi-squared tests), opioid use history, and knowledge of CBD were performed for all respondents, as well as for three distinct groups of respondents: Individuals who have never heard of CBD, individuals who have heard of CBD but never used CBD, and individuals who have used CBD. Additional descriptive statistics were performed on the group of individuals who used CBD to determine characteristics of their CBD use.

Individuals who have heard of but never used CBD, and individuals who have used CBD responded to statements regarding various aspects of CBD, such as its legality, social acceptance, and health implications. Responses were gathered using a five-point Likert scale ranging from “strongly disagree” to “strongly agree”. For quantitative analysis, these responses were encoded numerically from 1 (strongly agree) to 5 (strongly disagree).

For the inferential analysis, we employed ordinal logistic regression. This test was chosen to assess the statistical significance of differences in the Likert scale responses between individuals who have used CBD and those who have heard of CBD but have not used it while adjusting for potentially confounding variables (age and race). The resulting p-values from the test were used to determine whether the observed differences between the two groups' perceptions and use of CBD were statistically significant, with a conventional alpha level of 0.05 set for significance.

## Results

### Demographics

**Fig. 1:**
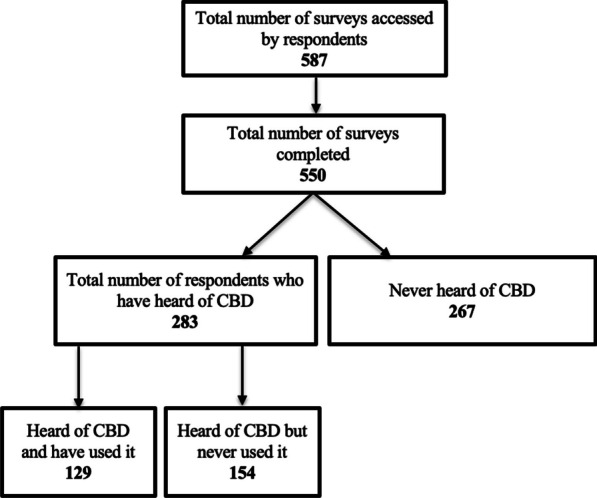
Breakdown of survey respondents by CBD knowledge and use

The survey was accessed by 587 respondents and 550 completed it. Participants were 51.88 ± 12.55 (Mean ± SD) years of age, with those who have used CBD being on average younger (43.8 ± 12.4) (*p* < 0.001). The majority were men (71.8%), non-white (69.3%), unemployed (55.3%) (Table 1), and had an OUD over 10 years (62.9%) and were managed with methadone (91.6%) (Table 2). Among survey completers, 267 never heard of CBD, while 283 (51.5%) had knowledge of CBD. Respondents who had heard of CBD learned of it through a variety of methods including from a friend or family member (108, 37.6%), the internet (71, 24.7%), social media (41, 14.3%), a sign at a store (18, 6.3%), a healthcare provider (25, 8.7%), or other (24, 8.4%). Among all survey completers, 129 (23%) reported a history of using CBD (Fig. 1).

**Table 1:** Sociodemographic characteristics of the study participants: all respondents and by CBD knowledge and use

Demographics	All respondents N = 550	Never heard of CBD N = 267	Heard of CBD but never used CBD N = 154	Have used CBD N = 129	t/x^2^	*p*
Age					4.049	< .001**
Mean (SD)	51.9 (12.6)	57 (10.2)	49.8 (12.4)	43.8 (12.4)		
Sex	N = (%)	N = (%)	N = (%)	N = (%)	1.250	.535
Female	154 (28%)	75 (28.1%)	44 (28.6%)	35 (27.1%)		
Male	395 (71.8%)	192 (71.9%)	110 (71.4%)	93 (72.1%)		
Gender non-conforming	1 (0.2%)	–	–	1 (0.8%)		
Race	N = (%)	N = (%)	N = (%)	N = (%)	13.775	.032*
American Indian or Alaska Native	18 (3.3%)	12 (4.5%)	4 (2.6%)	2 (1.6%)		
Asian	8 (1.5%)	2 (0.7%)	2 (1.3%)	4 (3.1%)		
Black or African American	131 (23.8%)	80 (30%)	37 (24%)	14 (10.9%)		
More than one race	45 (8.2%)	23 (8.6%)	8 (5.2%)	14 (10.9%)		
Native Hawaiian or Pacific Islander	2 (0.4%)	–	2 (1.3%)	–		
White or Caucasian	169 (30.7%)	43 (16.1%)	63 (40.9%)	63 (48.8%)		
Other	177 (32.1%)	107 (40.1%)	38 (24.7%)	32 (24.7%)		
Ethnicity	N = (%)	N = (%)	N = (%)	N = (%)	.003	.953
Hispanic/Latino	260 (43.7%)	141 (52.8%)	65 (42.2%)	54 (41.9%)		
Not Hispanic/Latino	290 (52.7%)	126 (47.2%)	89 (57.8%)	75 (58.1%)		
Education	N = (%)	N = (%)	N = (%)	N = (%)	18.151	.078
< High school	146 (26.5%)	82 (30.7%)	33 (21.3%)	17 (24.1%)		
High school diploma	175 (31.8%)	90 (33.6%)	39 (25.3%)	46 (35.7%)		
GED	130 (23.6%)	61 (22.8%)	42 (27.3%)	27 (20.9%)		
Associate’s degree	60 (10.9%)	21 (7.9%)	28 (18.2%)	11 (8.5%)		
Bachelor’s degree	29 (5.3%)	9 (3.4%)	9 (5.8%)	11 (8.5%)		
Master’s degree	6 (1.1%)	3 (1.1%)	2 (1.3%)	1 (0.8%)		
PhD or Advanced Professional degree	4 (0.7%)	1 (0.4%)	1 (0.6%)	2 (1.5%)		
Employment	N = (%)	N = (%)	N = (%)	N = (%)	6.805	.147
Full-time	62 (11.3%)	22 (8.2%)	25 (16.2%)	15 (11.6%)		
Part-time	43 (7.8%)	19 (7.1%)	14 (19.1%)	10 (7.8%)		
Self-employed	36 (6.5%)	11 (4.1%)	9 (5.8%)	16 (12.3%)		
Retired	105 (19.1%)	65 (24.3%)	26 (16.9%)	14 (10.9%)		
Unemployed	304 (55.3%)	150 (56.2%)	80 (51.9%)	74 (57.4%)		

Statistical analysis was performed comparing the ‘*Heard of CBD but Never Used CBD’* and ‘*Have Used CBD’* groups

**Table 2:** Opioid use characteristics of the study participants: all respondents and by CBD knowledge and use

Opioid Use	All respondents N = 550	Never heard of CBD N = 267	Heard of CBD but never used CBD N = 154	Have used CBD N = 129	X^2^	*p*
Number of years living with OUD	N = (%)	N = (%)	N = (%)	N = (%)	7.342	.119
< 1 year	11 (2%)	5 (1.9%)	4 (2.6%)	2 (1.6%)		
1–2 years	24 (4.4%)	12 (4.5%)	8 (5.2%)	4 (3.1%)		
2–5 years	68 (12.4%)	30 (11.2%)	14 (9.1%)	24 (18.6%)		
5–10 years	101 (18.4%)	47 (17.6%)	27 (17.5%)	27 (20.9%)		
> 10 years	346 (62.9%)	173 (64.8%)	101 (65.6%)	72 (55.8%)		
Duration of treatment for opioid use disorder	N = (%)	N = (%)	N = (%)	N = (%)	5.510	.239
< 1 year	70 (12.7%)	26 (9.7%)	27 (17.5%)	17 (13.2%)		
1–2 years	61 (11.1%)	22 (8.2%)	17 (11.0%)	22 (17.1%)		
2–5 years	113 (20.5%)	51 (19.1%)	31 (20.1%)	31 (24.0%)		
5–10 years	100 (18.2%)	46 (17.2%)	27 (17.5%)	27 (20.9%)		
> 10 years	206 (37.5%)	122 (45.7%)	52 (33.8%)	32 (24.8%)		
Medication for opioid use disorder	N = (%)	N = (%)	N = (%)	N = (%)	.925	.630
Methadone	504 (91.6%)	243 (91.0%)	144 (93.5%)	117 (90.7%)		
Buprenorphine	24 (4.4%)	14 (5.2%)	5 (3.2%)	5 (3.9%)		
Naltrexone	–	–	–	–		
No medication	22 (4.0%)	10 (3.7%)	5 (3.2%)	7 (5.4%)		
Opioid drug use other than methadone or buprenorphine?	N = (%)	N = (%)	N = (%)	N = (%)	4.701	.319
Daily	134 (24.4%)	58 (21.7%)	41 (26.6%)	35 (27.1%)		
Weekly	79 (14.4%)	37 (13.9%)	27 (17.5%)	15 (11.6%)		
Monthly	34 (6.2%)	19 (7.1%)	6 (3.9%)	9 (7.0%)		
Rarely	84 (15.3%)	28 (10.5%)	26 (16.9%)	30 (23.3%)		
Never	219 (39.8%)	125 (46.8%)	54 (35.1%)	40 (31.0%)		

Statistical analysis was performed comparing the ‘*Heard of CBD but Never Used CBD’* and ‘*Have Used CBD’* groups

### Reported use of CBD

**Fig. 2:**
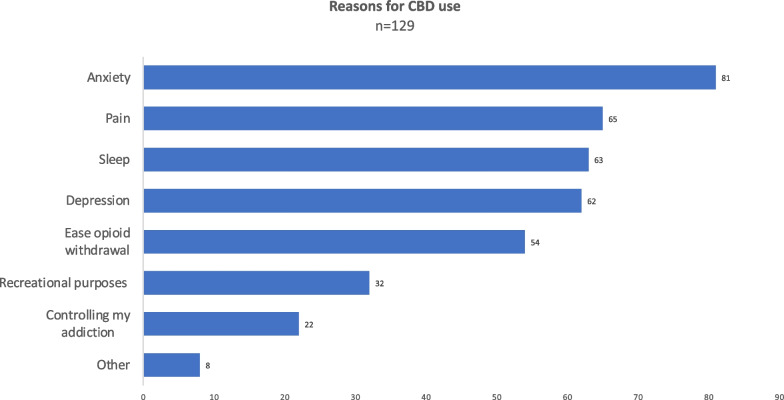
Respondent’s reasons for using CBD. Respondents were permitted to select as many as applied

**Fig. 3:**
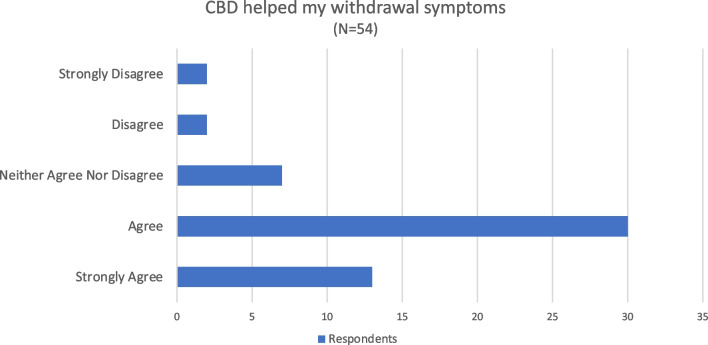
Perceptions of the efficacy of CBD on withdrawal symptoms among respondents who used CBD to ease opioid withdrawal symptoms

Of the 129 individuals who reported CBD use, it was obtained through several sources including: Smoke shops (51, 39.5%), medical cannabis dispensaries (26, 20.2%), the internet (15, 11.6%), gifts (10, 7.8%), pharmacies (7, 5.4%), convenience stores (4, 3.1%), or by other means (16, 12.4%). When consumed, it was in an oil form taken orally (43, 33.3%), an edible (32, 24.8%), vaped (38, 29.5%), applied topically (4, 3.1%), or other (12, 9.3%). CBD was used rarely (103, 79.8%) by respondents. However, some reported using CBD weekly (11, 8.5%), daily (14, 10.9%), or multiple times per day (1, 0.8%). The reason for use of CBD varied (Fig. 2) with respondents using it for anxiety (81, 62.8%), pain (65, 50.4%), sleep (63, 48.8%), depression (62, 48.1%), recreational purposes (32, 24.8%), or for other reasons (8, 6.2%). Of note, 22 (17.1%) respondents reported using CBD to control their addiction and 54 (41.9%) reported using CBD to ease opioid withdrawal symptoms. Among those who used CBD for withdrawal symptoms, 79.7% agreed or strongly agreed that CBD helped their withdrawal symptoms (Fig. 3).

An array of methods was used by survey respondents to describe how they determined a dose of CBD to take including: Product label (31, 24.0%), unsure (23, 17.8%), estimated (19, 14.7%), until I felt something (17, 13.2%), internet (16, 12.4%), advice from the cashier at the store (13, 10.1%), or healthcare provider recommended dose (10, 7.8%). Their last reported use of CBD ranged from more than 1 year ago (55, 42.6%), 1 month to 1 year ago (34, 26.4%), 1 week to 1 month ago (18, 14.0%), less than 1 week ago (10, 7.8%), to less than 24 h ago (12, 9.3%). Only 11 (8.5%) respondents reported side effects. Reported side effects included change in appetite (3), dry mouth (2), paranoia (2), feeling high (2), anxiety (1), upset stomach (1), and fatigue (1).

### Perceptions of CBD

**Fig. 4:**
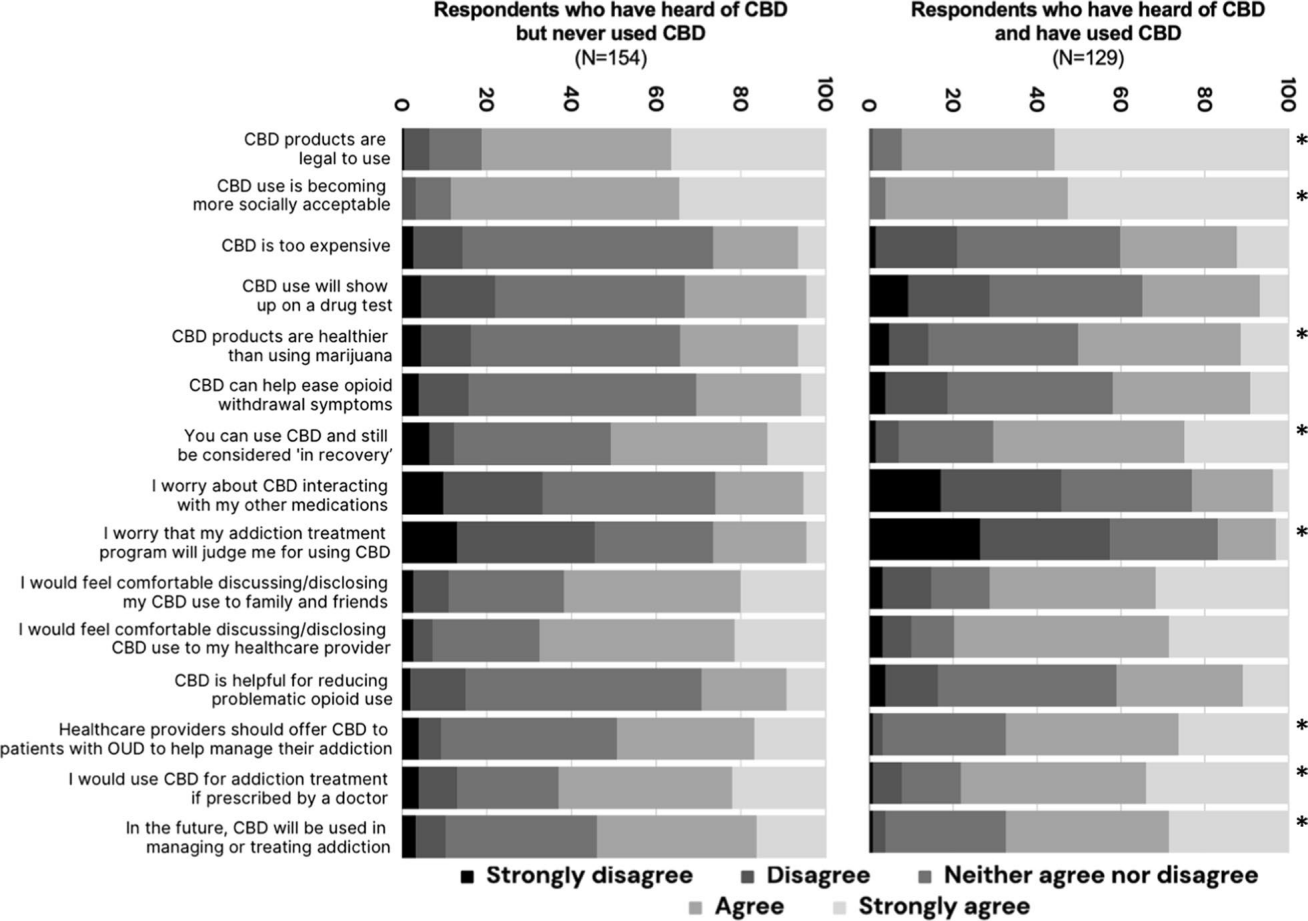
Stacked bar chart showing comparison of Likert scale answers to perception of CBD questions between respondents who have heard of CBD but never used it and respondents who have heard of CBD and have used CBD. *p < 0.05

Respondents demonstrated a generally positive stance towards CBD. These individuals felt that CBD is legal to use (244, 86.2%), consider CBD use as becoming more socially acceptable (260, 91.9%), and that one could use CBD and still be ‘in recovery’ (169, 59.7%). Most were comfortable discussing/disclosing CBD use to family/friends (187, 66%) or healthcare provider (207, 73.1%). While 144 (50.9%) respondents disagreed that their addiction treatment program would judge them for CBD use, 139 (49.2%) agreed or neither agreed nor disagreed with this statement indicating a potential stigma. One hundred sixty-three respondents (57.6%) believed healthcare providers should offer CBD to patients with OUD, and 198 (70%) would use CBD for OUD treatment if prescribed. One hundred seventy (60.1%) believed that CBD will be used for addiction treatment in the future. However, our analysis, presented in Table 3 and Fig. 4, revealed nuanced differences in perceptions between those who have used CBD and those who have not, with several key areas marked by statistically significant differences.

**Table 3:** Results of Likert scale questionnaire comparing results of respondents who have heard of CBD but have not used CBD and respondents who have used CBD

Statement	Group		Strongly disagree	Disagree	Neither agree nor disagree	Agree	Strongly agree	β	Odds ratio	95% CI	*p* value
CBD products are legal to use	Heard of CBD but have not used CBD	N	1	9	19	69	56	0.673	1.960	1.211–3.176	.006*
		%	0.6%	5.8%	12.3%	44.8%	36.4%				
	Have used CBD	N	0	1	9	47	72				
		%	0.0%	0.8%	7.0%	36.4%	55.8%				
CBD use is becoming more socially acceptable	Heard of CBD but have not used CBD	N	0	5	13	83	53	0.718	2.051	1.257–3.341	.004*
		%	0.0%	3.2%	8.4%	53.9%	34.4%				
	Have used CBD	N	0	0	5	56	68				
		%	0.0%	0.0%	5.0%	56.0%	68.0%				
CBD is too expensive	Heard of CBD but have not used CBD	N	4	18	91	31	10	0.273	1.314	0.836–2.064	.236
		%	2.6%	11.7%	59.1%	20.1%	6.5%				
	Have used CBD	N	2	25	50	36	16				
		%	1.6%	19.4%	38.8%	27.9%	12.4%				
CBD use will show up on a drug test	Heard of CBD but have not used CBD	N	7	27	69	44	7	0.078	1.081	0.696–1.680	.728
		%	4.5%	17.5%	44.8%	28.6%	4.5%				
	Have used CBD	N	12	25	47	36	9				
		%	12.0%	25.0%	47.0%	36.0%	9.0%				
CBD products are healthier than using marijuana	Heard of CBD but have not used CBD	N	7	18	76	43	10	0.562	1.754	1.120–2.747	.014*
		%	4.5%	11.7%	49.4%	27.9%	6.5%				
	Have used CBD	N	6	12	46	50	15				
		%	4.7%	9.3%	35.7%	38.8%	11.6%				
CBD can help ease opioid withdrawal symptoms	Heard of CBD but have not used CBD	N	6	18	83	38	9	0.118	1.125	0.717–1.763	.609
		%	3.9%	11.7%	53.9%	24.7%	5.8%				
	Have used CBD	N	5	19	51	42	12				
		%	5.0%	19.0%	51.0%	42.0%	12.0%				
You can use CBD and still be considered ‘in recovery’	Heard of CBD but have not used CBD	N	10	9	57	57	21	0.603	1.828	1.165– 2.865	.009*
		%	6.5%	5.8%	37.0%	37.0%	13.6%				
	Have used CBD	N	2	7	29	59	32				
		%	1.6%	5.4%	22.5%	45.7%	24.8%				
I worry about CBD interacting with my other medications	Heard of CBD but have not used CBD	N	15	36	63	32	8	− 0.292	0.747	0.482–1.155	.189
		%	9.7%	23.4%	40.9%	20.8%	5.2%				
	Have used CBD	N	22	37	40	25	5				
		%	22.0%	37.0%	40.0%	25.0%	5.0%				
I worry my addiction treatment program will judge me for using CBD	Heard of CBD but have not used CBD	N	20	50	43	34	7	− 0.520	0.595	0.384–0.920	.019*
		%	13.0%	32.5%	27.9%	22.1%	4.5%				
	Have used CBD	N	34	40	33	18	4				
		%	26.4%	31.0%	25.6%	14.0%	3.1%				
I would feel comfortable discussing/disclosing my CBD use to family and friends	Heard of CBD but have not used CBD	N	4	13	42	64	31	0.383	1.467	0.944–2.281	.089
		%	2.6%	8.4%	27.3%	41.6%	20.1%				
	Have used CBD	N	4	15	18	51	41				
		%	4.0%	15.0%	18.0%	51.0%	41.0%				
I would feel comfortable discussing/disclosing CBD use to my healthcare provider	Heard of CBD but have not used CBD	N	4	7	39	71	33	0.441	1.555	0.988–2.444	.056
		%	2.6%	4.5%	25.3%	46.1%	21.4%				
	Have used CBD	N	4	9	13	66	37				
		%	3.1%	7.0%	10.1%	51.2%	28.7%				
CBD is helpful for reducing problematic opioid use	Heard of CBD but have not used CBD	N	3	20	86	31	14	0.198	1.219	0.776–1.913	.391
		%	1.9%	13.0%	55.8%	20.1%	9.1%				
	Have used CBD	N	5	16	55	39	14				
		%	5.0%	16.0%	55.0%	39.0%	14.0%				
Healthcare providers should be offering CBD to patients with opioid use disorder to help manage their addiction	Heard of CBD but have not used CBD	N	6	8	64	50	26	0.726	2.067	1.318–3.236	.002*
		%	3.9%	5.2%	41.6%	32.5%	16.9%				
	Have used CBD	N	1	3	38	53	34				
		%	0.8%	2.3%	29.5%	41.1%	26.4%				
I would use CBD for addiction treatment if prescribed by a doctor	Heard of CBD but have not used CBD	N	6	14	37	63	34	0.618	1.856	1.185–2.907	.007*
		%	3.9%	9.1%	24.0%	40.9%	22.1%				
	Have used CBD	N	1	9	18	57	44				
		%	1.0%	9.0%	18.0%	57.0%	44.0%				
In the future, CBD will be used in managing or treating addiction	Heard of CBD but have not used CBD	N	5	11	55	58	25	0.613	1.846	1.181–2.887	.007*
		%	3.2%	7.1%	35.7%	37.7%	16.2%				
	Have used CBD	N	1	4	37	50	37				
		%	0.8%	3.1%	28.7%	38.8%	28.7%				

Survey responses were encoded numerically from 1 (strongly agree) to 5 (strongly disagree). *p < 0.05

Experience with CBD appeared to associate with perceptions of its legality and social acceptance. Respondents who used CBD were significantly more inclined to perceive CBD products as legal (β = 0.673, OR = 1.960, 95% CI [1.211, 3.176], *p* = .006) and to consider CBD use as becoming more accepted socially (β = 0.718, OR = 2.051, 95% CI [1.257, 3.341], *p* = .004). Users of CBD were also more likely to view CBD as a healthier alternative to marijuana (β = 0.562, OR = 1.754, 95% CI [1.120, 2.747], *p* = .014).

Participants who affirmed using CBD were significantly more likely to agree with the statement “You can use CBD and still be considered *'in recovery'”* (β = 0.603, OR = 1.828, 95% CI [1.165, 2.865], *p* = .09). However, concerns about judgment from addiction treatment programs regarding CBD use were evident in those who have heard of CBD but not used it (β = − 0.520, OR = 0.595, 95% CI [0.384, 0.920], *p* = 0019) signaling a potential stigma within treatment settings.

The respondents who used CBD also showed a strong belief in the potential of CBD to be prescribed by healthcare providers for managing opioid addiction (β = 0.726, OR = 2.067, 95% CI [1.318, 3.236], *p* = .002). Additionally, the belief that CBD will be used in managing or treating addiction in the future also differed significantly between the groups (β = 0.613, OR = 1.846, 95% CI [1.181, 2.887], *p* = .007), as well as willingness to use CBD if it were prescribed by a doctor (β = 0.618, OR = 1.856, 95% CI [1.185, 2.907], *p* = .007), with CBD users showing more positive attitude towards its potential role in addiction treatment.

## Discussion

There is an urgent need for novel strategies to mitigate the severity of the current opioid overdose crisis. This includes evaluating the use of cannabinoids as a potential harm reduction intervention [18]. Previous research has shown that CBD could reduce cravings for opioids in heroin-abstinent individuals and in patients with OUD maintained on buprenorphine [7, 19, 20]. However, these are small clinical trials and many questions remain as to whether OUD patients would be open to this cannabinoid as a potential treatment strategy.

Our survey results from individuals in treatment for OUD highlight a significant association between personal experience with CBD with more progressive views regarding its legality, social acceptance, and therapeutic potential. Individuals who have used CBD not only perceived it as a legal and socially acceptable substance, but were more inclined to believe it is healthier than traditional cannabis use. This group also showed an openness to incorporating CBD into their recovery process, despite a perceived stigma from addiction treatment programs. The stigmatization of using cannabis products for health purposes remains pervasive due to its ambiguous legal status and a lack of knowledge about the biological effects of cannabinoids [21–26].

Our findings show that some individuals in treatment for OUD are already using CBD as a harm reduction intervention to control their addiction or help ease symptoms associated with opioid withdrawal. Respondents also reported using CBD to manage health issues often associated with OUD, such as pain, anxiety, depression, and insomnia. While the reported side effects of CBD are minimal, they underscore the importance of an open dialogue about CBD use between healthcare providers and their patients. It is also essential to understand why a patient is using CBD and to educate them about potential adverse reactions, drug-drug interactions, and the risk of false positive urine toxicology results for tetrahydrocannabinol (THC).

The OTC availability of CBD, including oils, tinctures, capsules, smoke products, vape cartridges, balms, lotions, and gummies has led to widespread use [6]. Unfortunately OTC CBD products can be poor quality, with CBD and THC levels that do not match what is advertised [27]. Survey respondents most commonly learned about CBD from friends or family members rather than healthcare professionals, a finding that aligns with previous research [28]. Further, although survey respondents were willing to discuss their CBD use with healthcare providers, they were more likely to seek dosing advice from the internet, store cashiers, or simply guess, rather than consult their healthcare provider. This aligns with previous research which demonstrated that individuals frequently seek information about CBD from lay sources such as cannabis product retail staff, commonly known as “budtenders” [29].

There are many factors that may contribute to this gap in communication between patient and provider. Healthcare providers often lack knowledge about CBD and struggle to provide medical guidance to patients who are either already using CBD or considering its use [30, 31]. Additionally, the lack of medical guidelines on CBD, limited time during appointments, or belief that the patient would avoid disclosing CBD use, may prevent physicians from opening a dialogue with patients about the substance [31].

Positive attitudes regarding the potential of CBD as a medically-prescribed aid for managing opioid use highlight a pivotal role for patient-provider communication. The optimism reflected by the CBD user group about the future role of CBD in addiction treatment underscores the need for more robust clinical research to substantiate the therapeutic claims of CBD and its efficacy in the context of OUD treatment, as well as to develop clear guidelines to better inform healthcare providers when discussing CBD with their patients.

Historically, addiction treatment has involved coercion, social control, and stigmatization [32, 33]. Featuring the patient voice within the drug development process provides an avenue of empowerment to this historically marginalized and vulnerable group, creating the foundation for patient-centered and patient-driven treatments. By requesting input from patients early in the research process, we broaden the concept of allyship and shared decision making between physician and patient from beyond the clinic to the research sphere. In addition to improving the relationship between the medical field and this marginalized group, understanding patient perspectives has the potential to improve the drug development process and treatment outcomes [34].

### Limitations

There are several limitations to consider. The survey was collected between 2020 and 2023 during a heightened time of the COVID-19 pandemic. This made access to the addiction treatment clinics difficult due to safety concerns and there was a decrease in the frequency of patients going to the clinics. Another limitation was participant lack of access to smartphones or smart devices for completing the survey, thereby limiting the number of respondents. Moreover, this was a cross-sectional convenience sample rather than participants chosen by random selection. As such, the sample may not be representative of the broader population and there is a possibility that participants in the study could have answered the survey more than once. To mitigate these issues, the vast majority of surveys were conducted in person via direct recruitment from research personnel. Research staff alternated clinics and days to include a wider range of participants and better reflect the clinic populations for improved generalizability.

While the study specified that it was about CBD, there has been an unwavering growth of retail cannabis products. Some respondents may have confused CBD with marijuana or other cannabis products. Additionally, some products sold as CBD may also contain various amounts of THC or other cannabinoids. To try to address some of these concerns, we built gate questions and branching logic into the survey to focus on CBD. Additionally, for most surveys, research personnel were present at the site to answer clarifying questions. Lastly, while 129 respondents reported using CBD, the majority used it on an as-needed basis. Only 26 people (~ 5%) affirmed using CBD on a regular basis, but this low percentage is consistent with CBD use reported in individuals with substance use disorders [9].

## Conclusion

The current survey study provides valuable insights into the usage and perceptions of CBD among individuals in treatment for OUD. The findings reveal that some patients are already using CBD for a variety of reasons including anxiety, pain, sleep, depression, or as a harm reduction intervention to control their opioid use or minimize opioid withdrawal symptoms. This is often done without the knowledge of their healthcare providers. Respondents overall had a positive view of CBD suggesting a growing interest in its use as a potential adjunctive therapy for individuals with substance use disorders. The results also emphasize the importance of incorporating patient real-world experience and opinions into the development of future research and treatment approaches. By doing so, we can create more effective, patient-centered strategies that address the complexities of the opioid overdose crisis. Robust clinical research and clear medical guidelines are essential to harness the full potential of CBD as a harm reduction tool, ultimately improving outcomes for those struggling with OUD. 

### Supplementary Information

Additional file1.

## Data Availability

The datasets used and/or analyzed during the current study are available from the corresponding author on reasonable request.

## Abbreviations

CBD: Cannabidiol
OUD: Opioid use disorder
SUDs: Substance use disorders
THC: Tetrahydrocannabinol
