# Supplementary material for: Use and perceptions of Cannabidiol among individuals in treatment for opioid use disorder
Source: Harm Reduct J. 2024 Jul 17;21:135. doi: 10.1186/s12954-024-01051-5 (PMC11253449; doi:10.1186/s12954-024-01051-5)
Supplement: Supplementary file 1 — Additional file1. [file 12954_2024_1051_MOESM1_ESM.pdf]

Perceptions of Cannabidiol Among Patients with Opioid Use Disorder Survey

THE MOUNT SINAI HEALTH SYSTEM

ICAHN SCHOOL OF MEDICINE AT MOUNT SINAI

RESEARCH INFORMATION SHEET

Study ID #:
21-0

0682

Form Version Date: 5/7/21

Title: Perceptions of Cannabidiol Among Patients with Opioid Use Disorder

Researcher: Yasmin Hurd, PhD

1470 Madison Ave, NY NY 10029

212-824-93

14

The purpose of this research study is to study perceptions of cannabidiol among patients with opioid use disorder. You are being asked to take part in a research study because you have been diagnosed with opioid use disorder.

Being in a research study is completely voluntary. You can choose not to be in this research study. You can also say yes now, and change your mind later. Deciding not to be in the research study, now or later, will not affect your ability to receive medical care at Mount Sinai Medical Center.

If you agree to take part in this research, you will be asked to complete a short survey. Your participation in this study will take about 5 minutes. We expect that 500 people will take part in this research study.

You can choose not to answer any question you do not wish to answer. You can also choose to stop taking the survey at any time. You must be at least 18 years old to participate. If you are younger than 18 years old, please stop now.

The possible risks to you in taking part in this research are:

discomforts, including feeling uncomfortable, or having someone else find out that you were in a research study. Potential loss of confidentiality of data is also a risk.To protect your identity as a research subject, no identifiable information will be collected, the research data will not be stored with your name, the researcher(s) will not share your information with anyone. In any publication about this research, your name or other private information will not be used.

If you have any questions about this research, please contact the Researcher at (212)
58

5-4672. You can also call the Program for the Protection of Human Subjects Office at
212-824-8

200

This project was determined to be exempt from federal human subjects research regulations.

Version: 1.16.19

Date	______________________
Time	______________________
Do you give consent to participate in this survey?	◯ Yes ◯ No

Demographics

Age	__________________________________
Gender	◯ male<br>◯ female<br>◯ non-binary<br>◯ prefer to self-describe
please specify gender:<br><br>Please do not reveal any identifying information in this space (name, phone number, date of birth, etc...)	__________________________________
What is the highest level of education that you have completed?	◯ 6th Grade<br>◯ 7th Grade<br>◯ 8th Grade<br>◯ 9th Grade<br>◯ 10th Grade<br>◯ 11th Grade<br>◯ High School Diploma<br>◯ GED<br>◯ Associate's Degree<br>◯ Bachelor's Degree<br>◯ Master's Degree<br>◯ Ph.D. or Advanced Professional Degree
Ethnicity	◯ Hispanic/Latino<br>◯ Not Hispanic/Latino
How do you classify your race?	◯ White or Caucasian<br>◯ Black or African American<br>◯ American Indian or Alaska Native<br>◯ Asian<br>◯ Native Hawaiian or other Pacific Islander<br>◯ More than one race<br>◯ Other
If you selected "other," please specify:<br><br>Please do not reveal any identifying information in this space (name, phone number, date of birth, etc...)	__________________________________
Employment status	◯ full time<br>◯ part time<br>◯ unemployed<br>◯ retired<br>◯ self-employed

Opioid use history

Number of years living with an opioid use disorder:	◯ less than 1 year<br>◯ 1-2 years<br>◯ 2-5 years<br>◯ 5-10 years<br>◯ over 10 years<br>((years))
How long have you been in treatment for your opioid use disorder?	◯ less than 1 year<br>◯ 1-2 years<br>◯ 2-5 years<br>◯ 5-10 years<br>◯ over 10 years<br>((years))
Are you currently on medication for opioid use disorder?	◯ No current medications for addiction treatment<br>◯ Methadone<br>◯ Naltrexone (Vivitrol)<br>◯ Buprenorphine (Suboxone)
How often do you use opioid drugs other than methadone or Suboxone?	◯ daily<br>◯ weekly<br>◯ monthly<br>◯ rarely<br>◯ never
Have you ever heard of CBD or cannabidiol?	◯ Yes<br>◯ No

Thank You!

Thank you for taking the survey. Please click "Submit" on the next page.

CBD questionnaire

I learned about CBD from:	◯ friend or family member<br>◯ the internet<br>◯ social media<br>◯ sign at a store<br>◯ a healthcare provider<br>◯ other
If you selected "other" please specify:<br><br>Please do not reveal any identifying information in this space (name, phone number, date of birth, etc...)	__________________________________
Have you ever used CBD?	◯ Yes<br>◯ No
What was the route of CBD use	◯ oil taken orally<br>◯ edible<br>◯ vape<br>◯ topical<br>◯ other
If you selected "other", please specify:<br><br>Please do not reveal any identifying information in this space (name, phone number, date of birth, etc...)	__________________________________
The CBD was obtained from	◯ Internet<br>◯ smoke shop<br>◯ pharmacy<br>◯ medical cannabis dispensary<br>◯ gift<br>◯ convenient store<br>◯ other
If you selected "other", please specify:<br><br>Please do not reveal any identifying information in this space (name, phone number, date of birth, etc...)	__________________________________
How often do you use CBD?	◯ rarely<br>◯ weekly<br>◯ daily<br>◯ multiple times per day
I chose the dosage of the CBD by the following method	◯ internet<br>◯ product label recommendation<br>◯ estimate<br>◯ until I felt something<br>◯ healthcare provider recommended dose<br>◯ cashier at store<br>◯ unsure
Last use of CBD	◯ more than 1 year ago<br>◯ 1 month to 1 year ago<br>◯ 1 week to 1 month ago<br>◯ less than 1 week ago<br>◯ less than 24 hours ago

I use CBD to help with:<br><br>(choose as many as apply)	☐ anxiety<br>☐ depression<br>☐ sleep<br>☐ pain<br>☐ controlling my addiction<br>☐ I use it for recreational purposes<br>☐ other
If you selected "other" please specify:<br><br>Please do not reveal any identifying information in this space (name, phone number, date of birth, etc...)	__________________________________
I have used CBD to ease opioid withdrawal symptoms.	◯ Yes<br>◯ No
Have you experienced side effects of CBD?	◯ Yes<br>◯ No
What side effects have you experienced<br><br>(choose as many as apply)	☐ dry mouth<br>☐ upset stomach<br>☐ diarrhea<br>☐ feeling high<br>☐ change in appetite<br>☐ fatigue<br>☐ other
If you selected "other" please specify:<br><br>Please do not reveal any identifying information in this space (name, phone number, date of birth, etc...)	__________________________________
Please indicate whether you AGREE or DISAGREE with the following statements.	
CBD products are legal to use	◯ strongly agree<br>◯ agree<br>◯ neither agree nor disagree<br>◯ disagree<br>◯ strongly disagree
CBD helped my withdrawal symptoms.	◯ strongly agree<br>◯ agree<br>◯ neither agree nor disagree<br>◯ disagree<br>◯ strongly disagree
CBD use is becoming more socially acceptable	◯ strongly agree<br>◯ agree<br>◯ neither agree nor disagree<br>◯ disagree<br>◯ strongly disagree
CBD is too expensive	◯ strongly agree<br>◯ agree<br>◯ neither agree nor disagree<br>◯ disagree<br>◯ strongly disagree

CBD use will show up on a drug test	◯ strongly agree<br>◯ agree<br>◯ neither agree nor disagree<br>◯ disagree<br>◯ strongly disagree
CBD products are healthier than using marijuana	◯ strongly agree<br>◯ agree<br>◯ neither agree nor disagree<br>◯ disagree<br>◯ strongly disagree
CBD can help ease opioid withdrawal symptoms	◯ strongly agree<br>◯ agree<br>◯ neither agree nor disagree<br>◯ disagree<br>◯ strongly disagree
You can use CBD and still be considered "in recovery"	◯ strongly agree<br>◯ agree<br>◯ neither agree nor disagree<br>◯ disagree<br>◯ strongly disagree
I worry about CBD interacting with my other medications.	◯ strongly agree<br>◯ agree<br>◯ neither agree nor disagree<br>◯ disagree<br>◯ strongly disagree
I worry the my addiction treatment program will judge me for using CBD	◯ strongly agree<br>◯ agree<br>◯ neither agree nor disagree<br>◯ disagree<br>◯ strongly disagree
I would feel comfortable discussing/disclosing my CBD use to family and friends	◯ strongly agree<br>◯ agree<br>◯ neither agree nor disagree<br>◯ disagree<br>◯ strongly disagree
I would feel comfortable discussing/disclosing CBD use to my healthcare provider:	◯ strongly agree<br>◯ agree<br>◯ neither agree nor disagree<br>◯ disagree<br>◯ strongly disagree
CBD is helpful for reducing problematic opioid use	◯ strongly agree<br>◯ agree<br>◯ neither agree nor disagree<br>◯ disagree<br>◯ strongly disagree
Healthcare providers should be offering CBD to patients with opioid use disorder to help manage their addiction	◯ strongly agree<br>◯ agree<br>◯ neither agree nor disagree<br>◯ disagree<br>◯ strongly disagree

I would use CBD for addiction treatment if prescribed by a doctor	◯ strongly agree<br>◯ agree<br>◯ neither agree nor disagree<br>◯ disagree<br>◯ strongly disagree
In the future, CBD will be used in managing or treating addiction	◯ strongly agree<br>◯ agree<br>◯ neither agree nor disagree<br>◯ disagree<br>◯ strongly disagree
